# Peptide Drug Release Behavior from Biodegradable Temperature-Responsive Injectable Hydrogels Exhibiting Irreversible Gelation

**DOI:** 10.3390/gels3040038

**Published:** 2017-10-15

**Authors:** Kazuyuki Takata, Hiroki Takai, Yuta Yoshizaki, Takuya Nagata, Keisuke Kawahara, Yasuyuki Yoshida, Akinori Kuzuya, Yuichi Ohya

**Affiliations:** 1 Department of Chemistry and Materials Engineering, Faculty of Chemistry, Materials and Bioengineering, 3-3-35 Yamate, Suita, Osaka 564-8680, Japan; kazuyuki.takata@shionogi.co.jp (K.T.); k266872@kansai-u.ac.jp (H.T.); k161109@kansai-u.ac.jp (T.N.); kawaharakeisuke43@gmail.com (K.K.); k831414@gmail.com (Y.Y.); kuzuya@kansai-u.ac.jp (A.K.); 2 Organization for Research and Development of Innovative Science and Technology (ORDIST), Kansai University, Suita, Osaka 564-8680, Japan; y-yoshi@kansai-u.ac.jp; 3 Research Fellow of Japan Society for the promotion of Science, Kojimachi, Chiyoda-ku, Tokyo 102-0083, Japan

**Keywords:** injectable polymers, sustained release, sol-to-gel transition, hydrogel, peptide drug delivery

## Abstract

We investigated the release behavior of glucagon-like peptide-1 (GLP-1) from a biodegradable injectable polymer (IP) hydrogel. This hydrogel shows temperature-responsive irreversible gelation due to the covalent bond formation through a thiol-ene reaction. In vitro sustained release of GLP-1 from an irreversible IP formulation (**F(P1/D+PA_40_)**) was observed compared with a reversible (physical gelation) IP formulation (**F(P1)**). Moreover, pharmaceutically active levels of GLP-1 were maintained in blood after subcutaneous injection of the irreversible IP formulation into rats. This system should be useful for the minimally invasive sustained drug release of peptide drugs and other water-soluble bioactive reagents.

## 1. Introduction

Various temperature-responsive water-soluble polymers have been investigated for application in drug delivery systems (DDSs) [1,2,3,4,5,6,7,8,9]. Of these, several polymers in aqueous solution exhibit a temperature-responsive sol-to-gel transition between room temperature (r.t.) and body temperature and can thus be used as injectable polymer (IP) systems. Such polymer solutions can be mixed with water-soluble bioactive reagents such as proteins, peptides, or living cells before injection, and form a hydrogel entrapping these reagents at the injection site in the body. If the polymer can be hydrolyzed to low-molecular-weight compounds that can be metabolized or excreted from the body, such biodegradable IP systems could potentially act as implantable minimally invasive sustained drug release systems [10,11,12].

There have been many reports on drug release from hydrogels. The release rates of drugs from hydrogels are influenced by several factors, such as the hydrophilicity/hydrophobicity (solubility) and molecular weight of the drug, the degradation rates of the hydrogel, the mesh size of the network, and the diffusion constant of the drug in the hydrogel [13]. In general, temperature-responsive biodegradable IP hydrogels, once formed in the body, are likely to quickly revert to the sol state (typically in less than 24 h) at the injection site, where there is a large amount of body fluid [14]. This is because the gelation of such an IP system is caused by non-covalent (hydrophobic) interactions, and gel formation is an equilibrium process affected by local conditions such as concentration, pH, and temperature. This phenomenon may cause rapid disappearance of the hydrogel and more rapid release of bioactive agents than intended.

**Figure 1:**
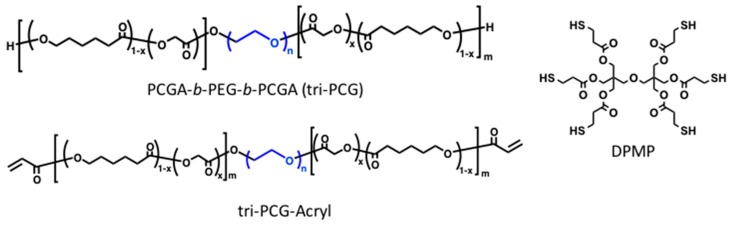
Structures of the polymers and polythiol used in this study.

To address this problem, we recently reported the generation of biodegradable temperature-triggered covalent gelation systems exhibiting longer and controllable durations of the gel state by using a “mixing strategy” [14,15,16]. We synthesized a tri-block copolymer of poly(caprolactone-*co*-glycolic acid) (PCGA), poly(ethylene glycol) (PEG), PCGA-*b*-PEG-*b*-PCGA (tri-PCG), and tri-PCG with acryloyl groups attached at both termini (tri-PCG-Acryl) (Figure 1). A mixture of tri-PCG-Acryl micelle solution and tri-PCG micelle solution containing dipentaerythritolhexakis(3-mercaptopropionate) (DPMP) (Figure 1) as a hydrophobic hexa-functional polythiol exhibited an irreversible sol-to-gel transition by covalent cross-linking using a bio-orthogonal Michael-addition-type thiol-ene reaction in response to a temperature increase [15,16]. The mixed micelle solution remained in the sol state just after mixing at r.t., but underwent gelation in response to a temperature increase. Once formed, the hydrogel stayed in the gel state even after cooling. In this system, the acryloyl groups at the copolymer termini and the thiol groups of DPMP existed separately in different micelles just after mixing. Covalent bond formation between the acryloyl and thiol groups occurred only upon sol-to-gel transition induced by a temperature increase, since this temperature rise induced inter-micellar aggregation due to hydrophobic interactions, resulting in a physically cross-linked hydrogel. During the aggregation process, the micelle cores fused and subsequently the thiol groups of DPMP in the tri-PCG micelle core covalently cross-linked with the acryloyl groups via the Michael-addition-type thiol-ene reaction. This system existed for a longer and controllable duration time in the gel state. The duration time of the gel state after subcutaneous injection in vivo could be altered easily from 1 day to more than 60 days simply by changing the mixing ratio of DPMP/tri-PCG and tri-PCG-Acryl [15].

Peptides are becoming increasingly important as drugs due to their activity, target specificity, tolerability, and availability. However, peptides must be administered via the parenteral route because their half-life in the body is extremely short and their oral absorption is poor. Consequently, when continuous exposure to the peptide drug is needed, continuous infusion or multiple injections are required to achieve therapeutic efficacy, which is inconvenient and distressing for patients. Thus, there is need to develop sustained release parenteral formulations, where a single injection allows the drug to be released over a period of weeks, months, or even years [17,18].

As described above, we previously developed a biodegradable temperature-triggered IP system exhibiting covalent gelation using a bio-orthogonal reaction and controllable duration times of the gel state. This system should have potential utility as a drug deposition method allowing the sustained release of peptide drugs. As far as we were aware, there has been no report on the drug release behavior using temperature-triggered covalent gelation system. In this study, we evaluated this IP system as a sustained peptide drug release device by studying the release behavior of a peptide from hydrogels prepared using our IP system. We chose glucagon-like peptide-1 (7-36 amide) (GLP-1) as the peptide model drug. GLP-1 holds promise for the treatment of type 2 diabetes but must be continuously infused or administered by multiple injections because of its extremely short half-life [19,20,21]. This is the first report on the drug release behavior using biodegradable temperature-responsive covalent gelation system exhibiting long-term release of peptide drugs.

## 2. Results and Discussion

Tri-PCGs (tri-PCG-1 and tri-PCG-2) and tri-PCG-Acryl were successfully synthesized according to the methods reported previously [15] (Schemes S1 and S2). Several characteristics of the polymers are shown in Supplementary Materials (Tables S1 and S2). The IP formulations were typically prepared by mixing DPMP-loaded tri-PCG micelles in phosphate buffered saline (PBS) (Solution A) and tri-PCG-Acryl micelles in PBS (Solution B) at a mixing ratio A/B = 3/2, with the weight content of tri-PCG-Acryl in the total polymer being 40%. This IP formulation is denoted as **F(P1/D+PA_40_)**, where P1, /D, and +PA_40_ denote the presence of tri-PCG-1 added, the presence of DPMP added, and the amount tri-PCG-Acryl added was 40 wt % in total polymers, respectively. The control formulation, prepared using only tri-PCG-1 or tri-PCG-Acryl, is denoted as **F(P1)** or **F(PA)**. The phase diagrams for tri-PCG-1 and tri-PCG-Acryl are shown in Supplementary Materials (Figure S1). Both these polymer solutions (25 wt %) and their mixtures adopted a gel state at 37 °C.

**Figure 2:**
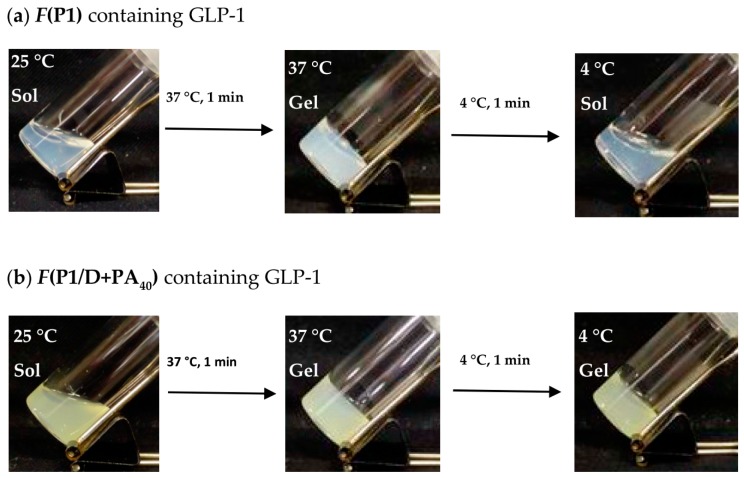
Photographs of (**a**) **F(P1)** hydrogel containing GLP-1 and (**b**) **F(P1/D+PA_40_)** hydrogel containing GLP-1 after heating at 37 °C for 1 min and subsequent cooling at 4 °C for 1 min.

The sol-to-gel transition behavior of the **F(P1/D+PA_40_)** and **F(P1)** formulations containing GLP-1 are shown in Figure 2. Both formulations showed a sol-to-gel transition in response to a temperature increase from 25 to 37 °C. The presence of GLP-1 had almost no effects on the sol-to-gel transitions of these formulations (Figure S2), and the transition temperatures were between 25 and 37 °C. After cooling to 4 °C, **F(P1)** containing GLP-1 adopted the sol state, showing that the sol-to-gel transition was reversible. In contrast, **F(P1/D+PA_40_)** containing GLP-1 remained in the gel state after cooling, showing that the sol-to-gel transition was irreversible. These behaviors are in accordance with our earlier observations in the absence of GLP-1 [15]. Consequently, the presence of GLP-1 had no effect on the phase transition irreversibility of the formulation.

**Figure 3:**
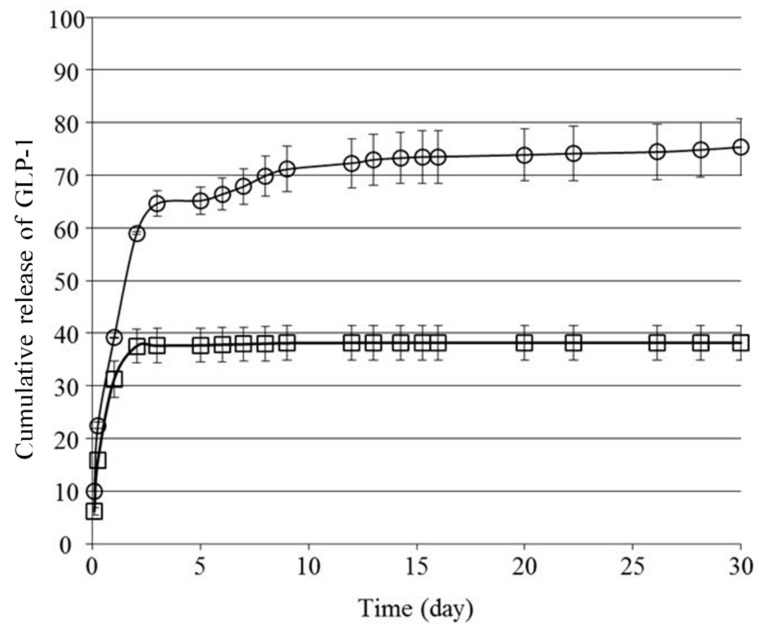
Cumulative release of GLP-1 (%) in vitro from **F(P1)** hydrogel containing GLP-1 (〇) and **F(P1/D+PA_40_)** hydrogel containing GLP-1 (**□**). The data are shown as the mean ± SD (*n* = 3).

**Figure 4:**
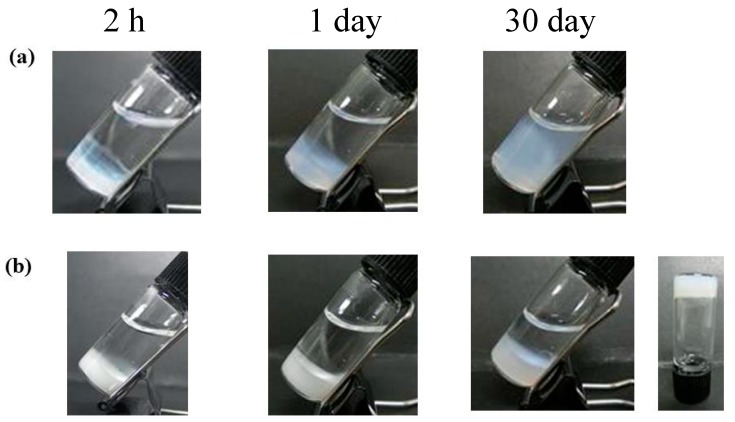
Photographs of (**a**) **F(P1)** hydrogel containing GLP-1 and (**b**) **F(P1/D+PA_40_)** hydrogel containing GLP-1 during the in vitro release test.

We next investigated the in vitro release behavior of GLP-1 from the formulations. Figure 3 shows the cumulative amount of GLP-1 released from **F(P1/D+PA_40_)** and **F(P1)** hydrogels at 37 °C in vitro. Both hydrogels showed rapid release of GLP-1 during the first two days, and the release rate from **F(P1)** was higher than that from **F(P1/D+PA_40_)**. On Day 2, the cumulative amount of GLP-1 released from **F(P1)** and **F(P1/D+PA_40_)** was about 59% and 38%, respectively. We previously reported that irreversible **F(P1/D+PA_x_)** IP hydrogels with higher DPMP and tri-PCG-Acryl content showed a lower swelling ratio compared with hydrogel without cross-linking (**F(P1)**) and compared with hydrogels with lower DPMP and tri-PCG-Acryl content. This lower swelling was due to the higher cross-linking densities of these gels [15]. The mesh size of a hydrogel increases due to swelling and cross-linking density correlates with the swelling ratio [22]. The main reason for the more rapid release of GLP-1 from **F(P1)** compared to **F(P1/D+PA_40_)** hydrogel is likely due to differences in the swelling ratios and diffusion coefficients of the two gels. Figure 4 shows photographs of the hydrogels taken during the release tests. The **F(P1)** hydrogel adopts a highly swollen state within 2 h and the formulation is essentially a sol after Day 1. By Day 2, the release of GLP-1 from **F(P1)** becomes gradual. As shown in Figure 4, although **F(P1)** reverts to the sol state completely by Day 30, 100% cumulative release is not attained. The GLP-1 molecule can adsorb onto the polymer chains by hydrophobic interactions [23], and GLP-1 may be unstable due to spontaneous hydrolysis; consequently, 100% of the GLP-1 molecules could not be detected by reversed-phase high-performance liquid chromatography (RP-HPLC) analysis. On the other hand, the release of GLP-1 from the **F(P1/D+PA_40_)** hydrogel became very slow after Day 2. As shown in Figure 4, **F(P1/D+PA_40_)** remained in the gel state even after 30 days. The storage modulus (G’) (4202 Pa) of **F(P1/D+PA_40_)** was larger than the loss modulus (G”) (326 Pa) and was similar to that of the sample on Day 0 (4696 Pa) (Table 1). As described above for **F(P1)** above, not all the GLP-1 molecules could be detected by RP-HPLC analysis. Regardless, some GLP-1 remained inside the hydrogel and was released very slowly.

**Figure 5:**
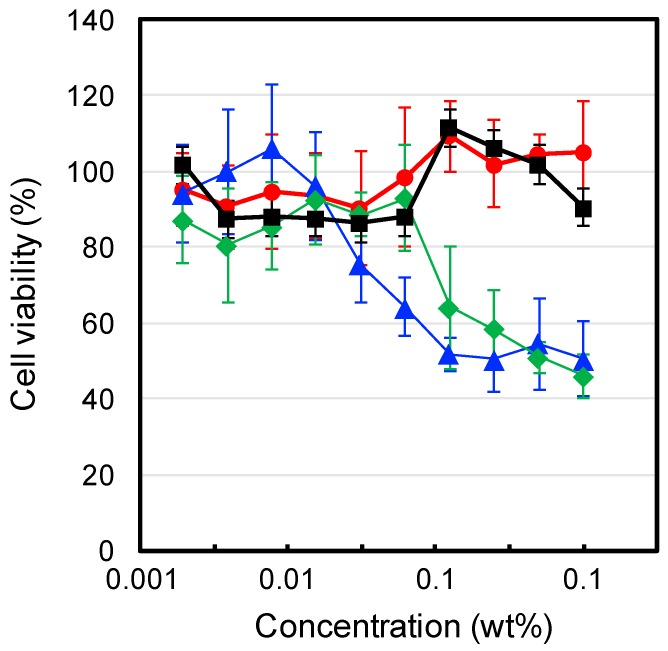
Cell viability of L929 fibroblast cells incubated in the presence of **F(P1)** (●), **F(P1/D)** (◆), **F(PA)** (▲) and **F(P1/D+PA50)** (■) in E-MEM containing 10% Fetal calf serum (FCS) at 37 °C for 21 h.

To evaluate the cytocompatibilities of the IP formulations, we investigated the cytotoxicity of each formulation and their components towards L929 mouse fibroblast cells. The experiments were under dilute conditions (<1 wt %), below the critical gelation concentration of each polymer. The results are shown in Figure 5. **F(P1)** (tri-PCG solution) exhibited no cytotoxicity over the concentration range tested. In contrast, **F(P1/D)** (tri-PCG micelle entrapping DPMP: solution (A) and **F(PA)** (tri-PCG-Acryl micelle solution: Solution (B) showed weak cytotoxicity at concentrations above 0.01 wt % and 0.1 wt %, respectively. Liu et al. reported that oligo-thiols such as dithiothreitol (DTT) are cytotoxic towards NIH/3T3 fibroblasts and rat bone marrow mesenchymal stem cells (BMSCs) [24]. Klouda et al. reported that polymers with multivalent acryloyl groups showed cytotoxicity [25]. Our results are therefore in agreement with these earlier reports. The thiol groups of DPMP in **F(P1/D)** and the acryloyl groups in **F(PA)** exhibited cytotoxicity, probably because of their interaction with the cell membrane. In contrast, the cytotoxicity of **F(P1/D+PA_50_)** was negligible, similar to that of **F(P1)**. These results suggest that the thiol groups of DPMP and the acryloyl groups of tri-PCG-Acryl reacted with each other, decreasing the number of both functional groups, and that the reaction products exhibited no cytotoxicity. Consequently, **F(P1/D+PA_50_)** showed no significant cytotoxicity.

**Figure 6:**
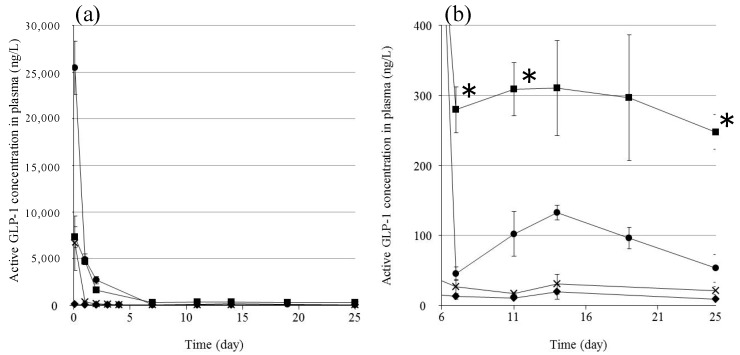
(**a**) Active GLP-1 concentration in plasma after subcutaneous injection for 25 days. (**b**) Magnification of the area between Days 6 and 25. GLP-1 solution (×), **F(P1)** containing GLP-1 (●), **F(P1/D+PA40)** containing GLP-1 (■), and **F(P1/D+PA40)** without GLP-1 (◆). The data are shown as the mean ± SD (*n* = 3–6). * *p* < 0.05 vs. **F(P1)** containing GLP-1.

**Figure 7:**
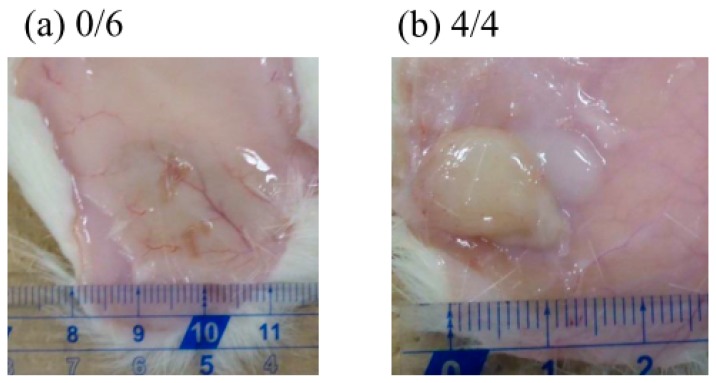
Photographs of hydrogels (**a**) **F(P1)**, (**b**) **F(P1/D+PA_40_)** 25 days after subcutaneous injection in rats. The values indicate the number of rats harboring hydrogel/all rats.

We investigated the in vivo plasma concentration of GLP-1 after subcutaneous injection of the formulations into rats (Figure 6). We used an ELISA system to detect only active GLP-1; inactivated GLP-1 is not detectable by this system. GPL-1 disappeared from the plasma very quickly following the injection of the GLP-1 solution: some GLP-1 was detected in the plasma after 2 h, but essentially none was detected after 1 day. This is as expected because the half-life of GLP-1 after subcutaneous injection is very short (t_1/2_ = 9 min) [21]. The injection of **F(P1)** formulations resulted in much higher plasma GLP-1 levels compared to the GLP-1 solution at 2 h, suggesting that the **F(P1)** hydrogel provided a delayed effect compared to the solution, allowing GLP-1 to be absorbed from the injection site and transferred to the blood circulation. The plasma GLP-1 level subsequently decreased rapidly, to 100 ng/L after 19 days, which is slightly higher than the level found in the control rats. The plasma GLP-1 level decreased to the control level 25 days after the injection of **F(P1)** hydrogel. These results are in relatively good agreement with the in vitro release tests (Figure 3): rapid, early release of GLP-1 from **F(P1)** hydrogel, then continuous sustained release from the sol state polymer chains acting as adsorbents in the subcutaneous space. **F(P1/D+PA_40_)** hydrogel also provided a relatively high plasma GLP-1 level 2 h after injection, similar to that of the GLP-1 solution and much lower than that of the **F(P1)** hydrogel. This result suggests that the initial burst release of GLP-1 was suppressed by the lower swelling of the gel resulting from covalent cross-linking, in good agreement with the in vitro results. Interestingly, although **F(P1/D+PA_40_)** hydrogel exhibited no detectable release of GLP-1 after three days in vitro, rats in the **F(P1/D+PA_40_)** group showed higher plasma GLP-1 levels (about 300 ng/L) between Day 7 and Day 25 compared with the **F(P1)** and control rats. These results suggest that the hydrogel network in **F(P1/D+PA_40_)** might be partially hydrolyzed by body fluids or by autocatalytic effects [26], resulting in the slow release of the GLP-1 entrapped in the hydrogel matrix in vivo. This speculation is supported by the results of a physical strength study (Table 1). The G’ value of subcutaneously implanted **F(P1/D+PA_40_)** hydrogel was 1147 Pa after 25 days, which is much lower than the G’ value obtained in vitro after 30 days’ incubation (4202 Pa), suggesting partial degradation. Kim et al. reported that the blood glucose level decreased significantly at a plasma GLP-1 level of around 200 ng/L [27]. Therefore, the plasma GLP-1 level (about 300 ng/L) obtained following the injection of **F(P1/D+PA_40_)** hydrogel containing GLP-1 should be sufficient to be pharmacologically active over a period of 25 days. Figure 7 shows photographs of the sites where the formulations were injected subcutaneously in rats 25 days earlier. All **F(P1)** hydrogel injected disappeared within 25 days, whereas all **F(P1/D+PA_40_)** hydrogel remained at the injection site after 25 days and was in the gel state (G’ (1147 Pa) was larger than G” (364 Pa); (Table 1)). We can, therefore, expect the continuous release of GLP-1 from **F(P1/D+PA_40_)** hydrogel even after 25 days.

## 3. Conclusions

We achieved long-term maintenance levels of plasma GLP-1 in vivo by using a biodegradable IP system that shows irreversible gelation due to covalent bond formation. Compared with a conventional physical gelation system, this irreversible hydrogel system exhibits a lower swelling ratio early in the drug release process, a suppressed initial burst, and sustained release of the encapsulated drug. Moreover, the system remained in the gel state for a longer time and gradually degraded after subcutaneous injection, which could help maintain a therapeutic blood drug level for over 1 month. This IP formulation did not exhibit severe cytotoxicity. Therefore, this irreversible IP hydrogel system holds promise for use in a minimally invasive sustained drug release system for hydrophilic compounds such as peptides and proteins. Especially, the IP system with GLP-1 can be a new effective therapeutic system for type 2 diabetes providing good quality of life of patients without frequent injections.

## 4. Materials and Methods

### 4.1. Materials

Tri-PCG-1 and tri-PCG-2 were synthesized by the ring-opening polymerization of ε-caprolactone (CL) glycolide (GL) in the presence of PEG (molecular weight (*MW*) = 1500 Da) according to the methods reported previously (Scheme S1) [15]. The *M*n of PCGA segments, the total *M*n, and the molar ratio of glycolic acid (GA) units to CL units in the tri-PCG-1 copolymer (CL/GA) were 1950, 5400 Da, and 3.4, respectively, and for tri-PCG-2 the values were 1250, 4000 Da, and 3.9, respectively (Table S1). Tri-PCG-Acryl was synthesized from tri-PCG-2 by the method described previously [15]. The total *M*n and the degree of substitution by acryloyl groups were 4200 Da and 91%, respectively (Table S2). GLP-1 (7–36 amide) was purchased from Aviva Systems Biology, Corp. (San Diego, CA, USA). DPMP was a gift from SC Organic Chemical Co., Ltd. (Osaka, Japan). Fetal calf serum (FCS) was obtained from Thermo Fisher Scientific (Waltham, MA, USA). Eagle’s minimum essential medium (E-MEM) was purchased from Nissui Pharmaceutical Co. (Tokyo, Japan). Mouse fibroblast NCTC clone 929 (L929) cells were obtained from the Health Science Research Resources Bank (HSRRB, Osaka, Japan). Spague-Dawley (SD) rats (7 weeks old, female, 180 g average body weight) were purchased from Japan SLC, Inc. (Hamamatsu, Japan). Water was purified using a Milli-Q (Merck Millipore, Billerica, MA, USA) system. All other reagents and organic solvents were of commercial grade and were used without further purification.

### 4.2. Preparation of the IP Formulations

The IP formulations were prepared as reported previously [15]. DPMP-loaded tri-PCG micelle solution (Solution A) was prepared as follows. Tri-PCG and DPMP were placed in a glass vial and dissolved with a small amount of acetone at r.t. The solution was dropped into pure water in a flask stirred at r.t. for 10 min, and then evaporated to remove the acetone. The aqueous solution was lyophilized to obtain powdery DPMP-loaded tri-PCG micelles. The powder was placed in a glass vial and a predetermined amount of PBS was added. After mixing with a vortex mixer for 1 min at r.t., the obtained suspension was heated to 65 °C and kept at 65 °C for 1 min, then mixed using a vortex mixer for 1 min at r.t. The glass vial was immersed in ice-cold water for 2 min and mixed with a vortex mixer for 1 min at r.t. These procedures were repeated until no insoluble particles were observed to give DPMP-loaded tri-PCG micelle solution (Solution A).

Tri-PCG-Acryl micelle solution (Solution B) was prepared as follows. Tri-PCG-Acryl was placed in a glass vial and PBS was added. After mixing with a vortex mixer for 1 min at r.t., the obtained suspension was heated to 65 °C and kept at 65 °C for 10 s, then further mixed using a vortex mixer for 1 min at r.t. The glass vial was immersed in ice-cold water for 2 min and mixed with a vortex mixer for 1 min at r.t. These procedures were repeated until no insoluble particles were observed to give tri-PCG-Acryl micelle solution (Solution B).

Finally, Solution A and Solution B were mixed at desired ratios to give IP formulations. The IP formulations are expressed as **F(P1/D+PA_x_)**, where P1, /D, and +PA_x_ denote the presence of tri-PCG-1 added, the presence of DPMP added, and the amount of tri-PCG-Acryl added was x wt % in total polymers.

### 4.3. In Vitro Release Test of GLP-1

IP formulations **F(P1/D+PA_40_)** containing GLP-1 were prepared as follows. A predetermined amount of GLP-1 was dissolved in Solution A (total polymer concentration = 26 wt %) by mixing and sonication, and the solution was then mixed with Solution B (total polymer concentration = 26 wt %). The pH was adjusted to 7.4 with 1N NaOH aqueous solution or HCl aqueous solution, and the total polymer concentration was adjusted to 25 wt % by the addition of PBS. The mixing ratio of solution A/solution B was 3/2, with the content of tri-PCG-Acryl in the total polymer being 40 wt %, **F(P1/D+PA_40_)**. As a control, IP formulation containing only tri-PCG-1 (without DPMP or tri-PCG-Acryl) and GLP-1 **F(P1)** was prepared by a similar method using only Solution A. The GLP-1 concentration in each IP formulation was 7.5 mg/mL.

Each formulation (200 µL) was placed in a glass vial and incubated at 37 °C for 30 min to obtain a hydrogel, then 1 mL of PBS as a release medium was gently added. At each sampling time, 0.6 mL of supernatant was removed and measured, and 0.6 mL fresh PBS was added to the vial, and the sample was then further incubated at 37 °C. The amount of GLP-1 in the sample solution was determined using a reversed-phase high-performance liquid chromatography (RP-HPLC) system (Waters, Milford, MA, USA) (column: Vydac 218TP54 (4.6 mm × 250 mm), eluent: acetonitrile containing 0.1% trifluoroacetic acid (TFA)/water containing 0.1% TFA, 1/4 to 4/1 linear gradient for 25 min; flow rate: 0.8 mL/min; detector: UV at 210 nm).

### 4.4. Cytotoxicity

The viability of L929 mouse fibroblast cells after incubation with each sample for 24 h was investigated using a WST-8 assay (Dojindo, Tokyo, Japan). L929 mouse fibroblast cells (100 μL, 2.5 × 10^3^ cells/well) in E-MEM containing 10% fetal bovine serum (FBS) were seeded in a 96-well microplate and cultured in a humidified atmosphere containing 5% CO_2_ at 37 °C. After preincubation for 24 h, all the medium supernatant was removed and added to 90 µL of fresh medium. Then, 10 µL of medium containing an IP formulation was added to each well and further incubated for 21 h. The medium supernatant was removed again from the wells, and the cells in the wells were washed with PBS twice. Thereafter, 90 μL of fresh medium and WST-8 reagent (10 μL) were added to the wells, and incubation was continued for a further 3 h. The microplates were read at 450 nm using a microplate reader. The average background absorbance from the control wells was subtracted from the sample data. The values for each sample were in the linear region of the standard curve for the WST-8 assay. Data are expressed as the means and SD (*n* = 6). Cell viability was calculated using the following equation:

Cell viability (%) = *N*t/*N*c × 100

where *N*t and *N*c are the number of cells with or without IP formulation after 21 h of incubation, respectively.

### 4.5. In Vivo Experiments

The **F(P1/D+PA_40_)** formulation (500 µL) containing GLP-1 was administrated by syringe with a 25 G needle subcutaneously in the back neck of a rat after anesthetizing with isoflurane. **F(P1)** containing GLP-1, **F(P1/D+PA_40_)** without GLP-1, and GLP-1 in PBS (pH 7.4) were used as controls. The volume of all samples was 500 µL, and the concentration of GLP-1 was 7.5 mg/mL.

At each sampling time (2 h–25 days), 250 µL blood samples were obtained from the tail vein using a blood collection tube (BD Microtainer with K2EDTA, Becton, Dickinson and Company, Franklin Lakes, NJ, USA). The blood samples were treated with 5 µL of dipeptidyl peptidase IV inhibitor (Merck Millipore, Billerica, MA, USA) and centrifuged (9100 g, 10 min, 4 °C) to obtain the plasma. The amount of active GLP-1 in the plasma was determined using an ELISA kit (GLP-1 active form assay kit, Immuno-Biological Laboratories Co., Ltd., Shizuoka, Japan). The results were expressed as mean ± SE (*n* = 3–6). Statistical comparisons were made using a Student’s *t*-test. A value of *p* < 0.05 was considered significant. Photographs of the rats injected with **F(P1)** without GLP-1 (top) and **F(P1/D+PA 40)** without GLP-1 just after injection, and after 1 day were shown in Figure S3 for references. These experiments followed the guidelines for animal experiments at Kansai University. The experiment was approved by the Ethical Committee for Animal Experiments of Faculty of Chemistry, Materials and Bioengineering, Kansai University (17 April, 2017, Identification number 1709).

### 4.6. Rheological Measurements

The physical properties of the formulations after soaking in PBS (release test) or after subcutaneous injection into rats were investigated at 37 °C by rheological measurements using a dynamic rheometer (Thermo HAAKE RS600, Thermo Fisher Scientific, Waltham, MA, USA). A solvent trap was used to prevent solvent vaporization. The diameter of the parallel plate was 35 mm, and the gap was 0.2 mm. The controlled stress and frequency were 0.4 Pa and 1.0 rad/s, respectively.

## Figures and Tables

**Table 1:** Physical properties of the hydrogels during release tests and in vivo experiments.

Formulation	In Vitro or Vivo	Day	G‘ (Pa)	G” (Pa)
**F(P1)**	in vitro	0	353	99
		30	N.D. ^1^	N.D. ^1^
	in vivo	25	N.D. ^2^	N.D. ^2^
**F(P1/D+PA_40_)**	in vitro	0	4694	569
		30	4202	326
	in vivo	25	1147	364

^1^ not detected because of being dissolved, ^2^ not detected because of disappearance.

## Supplementary Materials

The following are available online at www.mdpi.com/2310-2861/3/4/38/s1. Table S1: Characterization of PCGA-*b*-PEG-*b*-PCGA triblock copolymers (tri-PCGs); Table S2: Characterization of tri-PCG-Acryl; Scheme S1: Synthesis of PCGA-b-PEG-b-PCGA triblock copolymer (tri-PCG); Scheme S2: Synthesis of tri-PCG-Acryl; Figure S1: Phase diagrams of (**a**) tri-PCG and (**b**) tri-PCG-Acryl. ●: sol; ●: gel; ●: sol (syneresis). The gelation temperature (*T*_gel_) of each concentration is indicated; Figure S2: Comparison of the gelation temperature in the presence or absence of GLP-1 for (**a**) tri-PCG and (**b**) tri-PCG-Acryl. ●: sol; ●: gel; ●: sol (syneresis). The polymer concentration = 25 wt%. The gelation temperature (*T*_gel_) of each sample is indicated; Figure S3: Photographs of the rats injected with **F(P1)** without GLP-1 (top) and **F(P1/D+PA 40)** without GLP-1 just after injection, and after 1 day.
