# Supplementary material for: Peptide Drug Release Behavior from Biodegradable Temperature-Responsive Injectable Hydrogels Exhibiting Irreversible Gelation
Source: Gels. 2017 Oct 15;3(4):38. doi: 10.3390/gels3040038 (PMC6318690; doi:10.3390/gels3040038)
Supplement: Supplementary file 1 [file gels-03-00038-s001.pdf]

Table S1. Characterization of PCGA-*b*-PEG-*b*-PCGA triblock copolymers (tri-PCGs)

Code	DP of CL[a)]	DP of GA[b)]	CL/GA[c)]	M_n (Da)[d)]	M_w (Da)[e)]	M_w/M_n[e)]
tri-PCG-1	14.6	4.3	3.4	5,400	6,900	1.38
tri-PCG-2	9.9	2.6	3.9	4,000	4,800	1.38

a) The degree of polymerization of the ε-caprolactone unit in a PCGA segment was calculated using ^{1}H-NMR.
b) The degree of polymerization of a glycolic acid unit in a PCGA segment was calculated using ^{1}H-NMR.
c) Molar ratio of CL/GA in a PCGA segment estimated using ^{1}H-NMR
d) Number-average of the molecular weight estimated using ^{1}H-NMR.
e) Weight-average of the molecular weight and the polydispersity index estimated using SEC.

Table S2. Characterization of tri-PCG-Acryl

Code	M_n (Da)[a)]	M_w (Da)[b)]	M_w/M_n[b)]	DS (%)[c)]
tri-PCG-Acryl	4,200	5,100	1.40	91

a) Number-average of the molecular weight estimated using ^{1}H-NMR.
b) Weight-average of the molecular weight and the polydispersity index estimated using SEC.
c) Degree of substitution of the acryloyl group calculated using ^{1}H-NMR.

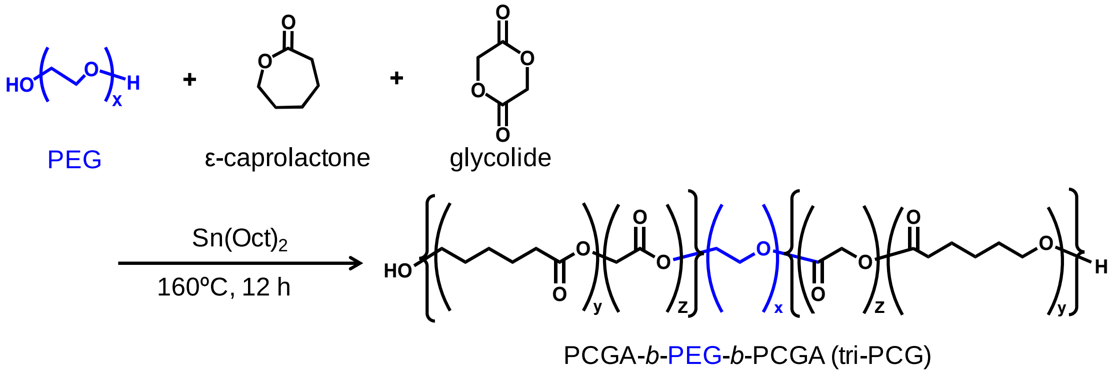

Scheme S1. Synthesis of PCGA-*b*-PEG-*b*-PCGA triblock copolymer (tri-PCG).

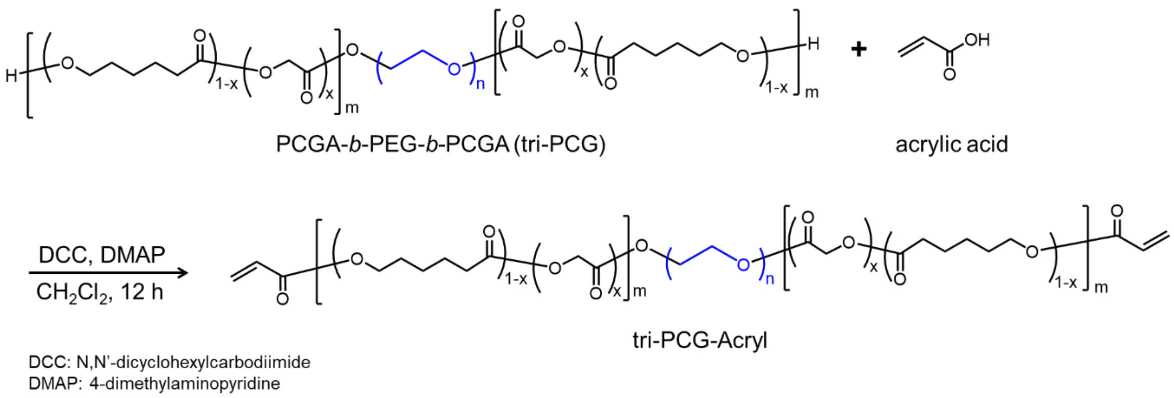

Scheme S2 Synthesis of tri-PCG-Acryl

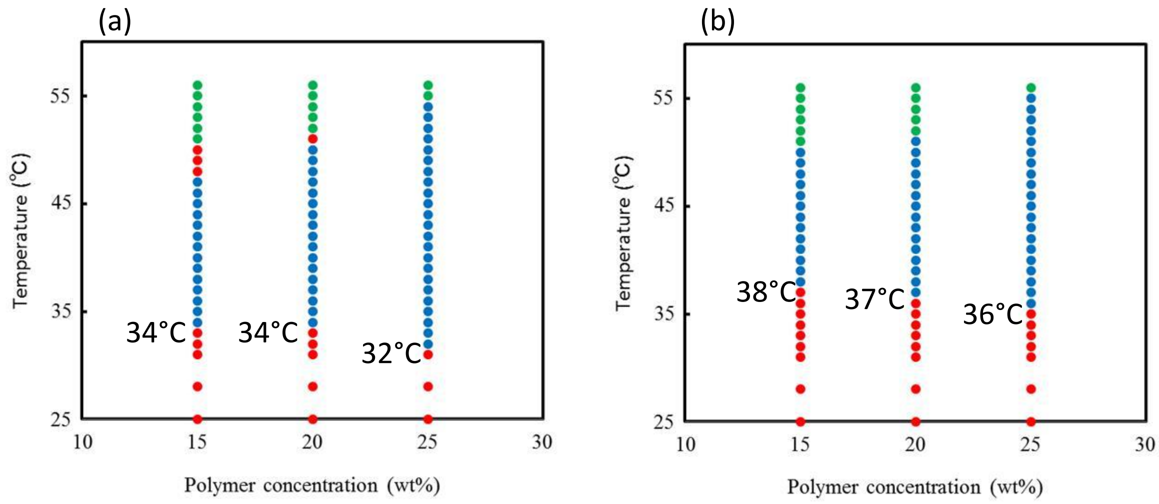

Figure S1. Phase diagrams of (a) tri-PCG and (b) tri-PCG-Acryl. ●: sol, ●: gel, ●: sol (syneresis). The gelation temperature (T_{gel}) of each concentration is indicated.

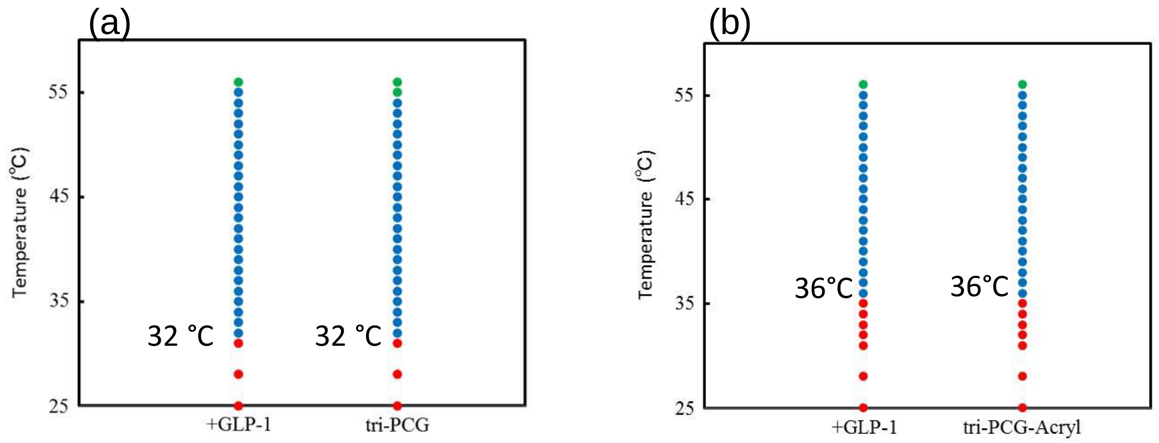

Figure S2. Comparison of the gelation temperature in the presence or absence of GLP-1 for (a) tri-PCG and (b) tri-PCG-Acryl. ●: sol, ●: gel, ●: sol (syneresis). The polymer concentration = 25 wt%. The gelation temperature (T_{gel}) of each sample is indicated.

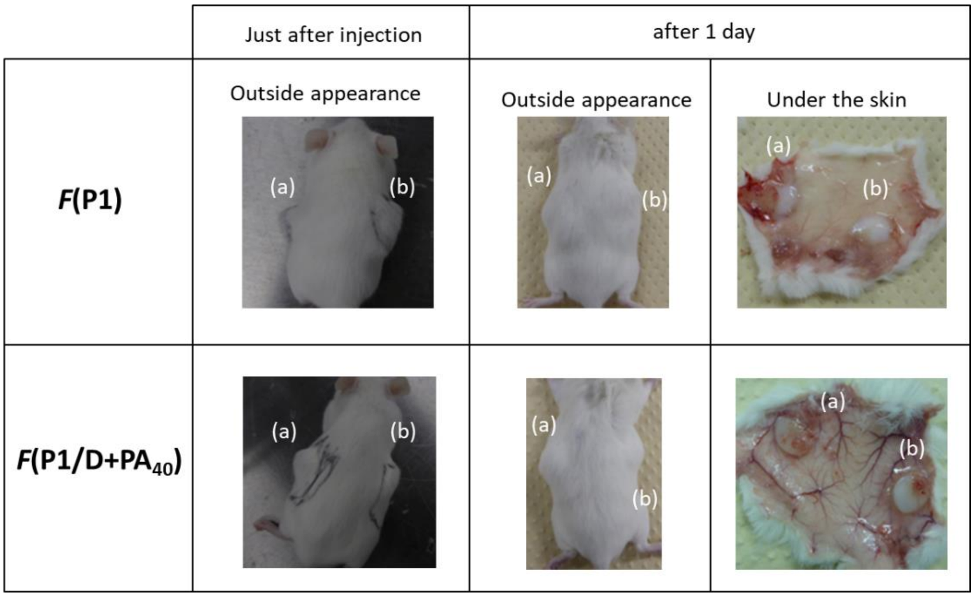

Figure S3. Photographs of the rats injected with ***F*(P1)** without GLP-1 (top) and ***F*(P1/D+PA$_{40}$)** without GLP-1 just after injection, and after 1 day.
